# In-Cell Western Assays to Evaluate Hantaan Virus Replication as a Novel Approach to Screen Antiviral Molecules and Detect Neutralizing Antibody Titers

**DOI:** 10.3389/fcimb.2017.00269

**Published:** 2017-06-20

**Authors:** Hong-Wei Ma, Wei Ye, He-Song Chen, Tie-Jian Nie, Lin-Feng Cheng, Liang Zhang, Pei-Jun Han, Xing-An Wu, Zhi-Kai Xu, Ying-Feng Lei, Fang-Lin Zhang

**Affiliations:** ^1^Department of Microbiology, Fourth Military Medical UniversityXi'an, China; ^2^Department of Neurosurgery, Tangdu Hospital, Fourth Military Medical UniversityXi'an, China

**Keywords:** hantaan virus, in-cell western assays, DDX21, DDX60, interferon, neutralizing antibody, disease severity

## Abstract

Hantaviruses encompass rodent-borne zoonotic pathogens that cause severe hemorrhagic fever disease with high mortality rates in humans. Detection of infectious virus titer lays a solid foundation for virology and immunology researches. Canonical methods to assess viral titers rely on visible cytopathic effects (CPE), but Hantaan virus (HTNV, the prototype hantavirus) maintains a relatively sluggish life cycle and does not produce CPE in cell culture. Here, an in-cell Western (ICW) assay was utilized to rapidly measure the expression of viral proteins in infected cells and to establish a novel approach to detect viral titers. Compared with classical approaches, the ICW assay is accurate and time- and cost-effective. Furthermore, the ICW assay provided a high-throughput platform to screen and identify antiviral molecules. Potential antiviral roles of several DExD/H box helicase family members were investigated using the ICW assay, and the results indicated that DDX21 and DDX60 reinforced IFN responses and exerted anti-hantaviral effects, whereas DDX50 probably promoted HTNV replication. Additionally, the ICW assay was also applied to assess NAb titers in patients and vaccine recipients. Patients with prompt production of NAbs tended to have favorable disease outcomes. Modest NAb titers were found in vaccinees, indicating that current vaccines still require improvements as they cannot prime host humoral immunity with high efficiency. Taken together, our results indicate that the use of the ICW assay to evaluate non-CPE Hantaan virus titer demonstrates a significant improvement over current infectivity approaches and a novel technique to screen antiviral molecules and detect NAb efficacies.

## Introduction

Hantaviruses, which constitute one of five genera forming the *Bunyaviridae* family, are enveloped zoonotic viruses with a negative sense single-strand RNA (ssRNA) genome. The hantaviral tripartite genome consists of the S, M, and L segments, which encode the nucleoprotein (NP), glycoprotein (GP, which is post-translationally cleaved into the N-terminal Gn and C-terminal Gc components), and viral RNA-dependent RNA polymerase (RdRp), respectively. Hantaviruses are transmitted to humans by persistently infected rodents. Following infection, the virus targets host vascular endothelial cells and causes increased vascular permeability and serious immune injury. Depending on the virus type, hantaviruses give rise to hemorrhagic fever with renal syndrome (HFRS) or pulmonary syndrome (HPS; Guardado-Calvo et al., 2016). A total of 150,000–200,000 hantavirus infection cases are reported annually worldwide, with mortality rates of 15% for HFRS and 50% for HPS during the natural infection process (Hussein et al., 2012). Notably, Chinese HFRS patients account for ~90% of the total global cases each year. Over the past 60 years, almost 1.7 million cases and 47,000 deaths have been reported in China (Jiang et al., 2016). Hantaan virus (HTNV), which is the prototype hantavirus discovered in the early 1950s during the Korean war (D, 1954), is the major causative agent of HFRS in China. The clinical course of HFRS typically proceeds through five phases (the febrile, hypotensive shock, oliguric, diuretic and convalescent stages). To date, neither effective therapeutic drugs nor FDA licensed prophylactic vaccines against HTNV infection are available. Rapid detection of viral titer is indispensable for developing therapeutic drugs or prophylactic strategies against HTNV infection.

The infectious virus titer has been conventionally measured by plaque assays, which are based on virus-induced cytopathic effects (CPE). Nevertheless, one significant characteristic of hantaviruses is that their replication in mammalian cell culture tends to be slow and non-lytic (McCaughey et al., 1999). Several traditional methods have been developed to detect hantavirus replication, such as the improved plaque formation test (McCaughey et al., 1999), enzyme-labeled immunosorbent assay (ELISA; Cheng et al., 2014), quantitative real-time RT-PCR (qRT-PCR; Machado et al., 2013), immunofluorescence assay (IFA; Xu et al., 2002; Jin et al., 2012) and flow cytometry (FCM; Barriga et al., 2013). The improved plaque formation test is dependent on the low pH-induced cytopathic effects of hantavirus but is time-consuming and has low reproducibility. The most widely adopted approach to test hantavirus titers (especially for HTNV) is the TCID50 (50% tissue culture infective dose) calculation using ELISA as previously reported by our group (Xu et al., 2002; Cheng et al., 2014; Jiang et al., 2015; Ye et al., 2015a,b; Ying et al., 2016); however, virus propagation in Vero E6 cells takes at least 10 days. All the reported detective measurements have insurmountably objective drawbacks, such as high demanding experimental conditions for qRT-PCR and expensive apparatus and labware for FCM, which limits their applicability (Wan et al., 2010).

To narrow this gap, in-cell Western (ICW) assays have been applied to monitor hantavirus replication kinetics and assess viral titers. The ICW assay is a cell-based technique for intracellular protein detection that is characterized by high rapidity, accuracy, sensitivity, and reproducibility (Egorina et al., 2006). The ICW procedure mainly includes cell fixation, a target protein combined with primary antibodies and subsequent infrared-labeled secondary antibodies (Mukherjee et al., 2013). The expression level of the target protein is determined using the relevant immunofluorescent value (intensity ratio; immunofluorescent intensity of the target protein vs. an endogenous protein). To date, the ICW assay has been exploited largely for the quantitative analysis of cellular signaling pathways (Schnaiter et al., 2014; Boveia and Schutz-Geschwender, 2015), whereas its application in the detection viral replication has been scarcely reported.

In the present study, the ICW assay was used to detect HTNV NP expression and monitor viral replication kinetics, based on which viral and NAb titers were evaluated. Compared with other classical approaches, ICW assays exhibit high convenience, celerity and accuracy. To note, the ICW assay offered a high-throughput platform for screening anti-hantaviral molecules and assessing NAb titers. Here, using digital gene expression (DGE) analysis, multiple DExD/H box helicase family members were found to be upregulated in human umbilical vascular endothelial cells (HUVECs) after HTNV infection, some of which were reported to exert antiviral effects (Chen et al., 2014). Through ICW-dependent filtration, we found that DDX21 and DDX60 suppressed HTNV infection in an IFN-dependent manner, whereas DDX50 was a positive regulator of HTNV replication. Additionally, the NAb titers in sera from 64 HFRS patients and 16 vaccinated individuals were assessed by ICW. NAb production in the patients was correlated with the disease course and severity. Poor NAb titers in the vaccinees suggested that current inactivated HTNV vaccines could not activate robust humoral immunity in hosts. Taken together, our results indicate that the established ICW assay used to qualify HTNV replication provides a rapid and high-throughput approach to screen anti-hantaviral molecules and assess NAb titers, which will aid in the development of antiviral drugs and evaluations of vaccine efficacy.

## Materials and methods

### Virus and cells

HTNV strain 76–118 was preserved in our lab, and all related experiments were approved by the FMMU biosafety committee and performed in a biosafety level 2 (BSL-2) facility. As a control, cells were incubated with a culture supernatant from uninfected Vero E6 cells (Vero C1008, ATCC, CRL 1586), which were referred to as mock-infected cells. HUVECs purchased from ScienCell Research Laboratories (Carlsbad, CA, USA) and cultured in ECM BulletKit (ScienCell Research Laboratories) were used for the HTNV infection study to screen anti-hantaviral molecules or drugs. A549 cells purchased from ATCC (CRM-CCL-185, Manassas, VA, USA) and grown in Dulbecco's modified Eagle's medium (DMEM; HyClone, Logan, UT, USA) supplemented with 10% fetal bovine serum (FBS; Gibco, Grand Island, NY, USA) were prepared for the HTNV infection study to assess the viral and NAb titers. Vero E6 cells maintained in DMEM (HyClone) with 10% FBS (Gibco) were used to propagate HTNV, assess the viral and NAb titers, and identify the antiviral activities. HEK293 cells (293T, ATCC, CRL 1586) grown in DMEM (HyClone) with 10% FBS (Gibco) were prepared for lentiviral packaging and propagation.

### Generation of monoclonal antibodies (mAbs)

Previously identified hybridoma cells were injected intraperitoneally (i.p.) into 9-week-old pristane-primed BALB/c mice (Medical Experimental Animal Center, Xian, China). Ascitic fluid was collected as the mAb source. The mAb 1A8 (mouse IgG1) targeted the HTNV NP, whereas 3D8 and 3G1 recognized the HTNV GP (Xu et al., 2002). Mouse ascitic fluid generated by i.p. inoculation of Sp2/0 cells was used as a control. The mAbs were purified by saturated ammonium sulfate precipitation and quantified with a Bicinchoninic Acid (BCA) Kit (Sigma-Aldrich, St. Louis, MO, USA) according to the manufacturer's instructions. Then, the mAbs were diluted to a final concentration of 10 μg/μl for application in the ICW assay. Experiments with mice were conducted in compliance with a protocol approved by the Institutional Animal Care and Use Committee of FMMU based on the Ethical Principles in Animal Experimentation.

### In-cell western (ICW) assay

The ICW assay was performed using the Odyssey Imaging System (LI-COR Biosciences, Lincoln, NE, USA) according to the manufacturer's instructions. Briefly, cells grown in 96-well plates (Falcon™, Cat# 161093, BD Biosciences, San Jose, CA, USA; Nunc™, Cat# 161093, Thermo Fisher Scientific, Waltham, MA, USA) until they reached 60–70% confluency were either mock infected or infected with HTNV at a given MOI and then fixed with 4% paraformaldehyde (PFA) at selected time intervals post-infection for the ICW assay. Then, the cells were permeabilized with 0.5% Triton X-100 for 15 min at room temperature (RT) and blocked with LI-COR Odyssey Blocking Solution (LI-COR Biosciences) for 30 min. The cells were incubated at 4°C overnight with 1A8, 3D8, or 3G1 pre-mixed with a rabbit IgG antibody against β-actin (1:200 dilution, Protein Tech Inc., Wuhan, China). After five washes with DPBS (HyClone), the cells were stained with a goat anti-mouse IgG IRDye™ 800 antibody (1:5,000 dilution, LI-COR Biosciences) and a goat anti-rabbit IgG IRDye™ 680 antibody (1:5,000 dilution, LI-COR Biosciences) at RT for 2 h. The microplates, which could be preserved from light at 4°C for at least 6 months, were scanned with the Odyssey CLx Infrared Imaging System (LI-COR Biosciences), and the integrated fluorescence intensities representing the protein expression levels were acquired using the software provided with the imager station (Odyssey Software Version 3.0, LI-COR Biosciences). The relative amount of NP protein was obtained by normalizing to endogenous β-actin in all experiments.

For HTNV titer measurement, A549 cells grown in microplates until they reached 60–70% confluency were incubated with gradient diluted HTNV, which was propagated in a BALB/c mouse brain and then in Vero E6 cells, at 37°C for 90 min. Then, the A549 cells were washed with DPBS (HyClone) and cultured in DMEM with 10% FBS. At 48 h post-infection, the ICW assay were performed to detect the amount of HTNV NP; positive/negative (P/N) responses >2.1 were considered significant. The viral titer was calculated as the TCID50 using the Reed and Muench formula.

To screen and confirm the identified antiviral molecules, HUVECs or Vero E6 cells in microplates were infected with the relevant lentiviruses at an MOI of 100. The medium was replaced 12 h later, and after another 12 h the cells were infected with hTNV at an MOI of 0.1. At 48 h post-infection, the ICW assay was performed to detect the amount of hTNV NP. The overexpression efficiency of IFITM1, IFITM2, IFITM3, Mx1 and Mx2 (IFITM-lentiviruses were purchased from GENECHEM, Shanghai, China, and Mx-lentiviruses were packaged and verified by our lab) was determined with the ICW assay using mouse monoclonal antibodies against the corresponding molecules (Protein Tech Inc.) at 24 h post-lentiviral infection. The overexpression efficiency of DDX3, DDX5, DDX6, DDX21, DDX50, and DDX60 (lentiviruses packaged and verified by our lab) was examined by detecting ZsGreen fluorescence in the cells at 24 h post-lentiviral infection.

To assess the NAb titers, 500-fold of the TCID50 was adequately mixed with 3D8, 3G1, or patient sera and incubated at 37°C for 90 min. A549 cells grown in microplates until they reached 60–70% confluency were incubated with the mixture at 37°C for 90 min. Then, the cells were washed with DPBS (HyClone) two times and incubated with DMEM with 10% FBS. Forty hours later, the A549 cells were harvested for the ICW assay to assess NP expression.

### Quantitative real-time PCR (qRT-PCR) analysis

HUVECs and A549 cells grown in 24-well plates were infected with HTNV. RNA was extracted using RNAiso (TaKaRa Biotechnology Co., Dalian, China) at different time points. cDNA was synthesized with the PrimeScript RT Master Mix (TaKaRa) according to the manufacturer's instructions. Quantitative real-time PCR (qRT-PCR) was performed using SYBR Premix Ex Taq II (Takara) with the Roche LightCycler 96 (Roche, Basel, Switzerland). β-actin was used as the reference gene. The oligonucleotide primers used for the qRT-PCR were synthetized at Sangon (Shanghai, China); the sequences are listed in Table 1. The HTNV loads were calculated as previously reported (Yi et al., 2013).

**Table 1:** Oligonucleotide primers and sequences.

**Target Genes**	**Primer sequences**
β-actin-F	5′-GCTACGTCGCCCTGGACTTC-3′
β-actin-R	5′-GTCATAGTCCGCCTAGAAGC-3′
HTNV-S-F	5′-GCCTGGAGACCATCTGAAAG-3′
HTNV-S-R	5′-AGTATCGGGACGACAAAGGA-3′
IFITM1-F	5′-ACTCCGTGAAGTCTAGGGACA-3′
IFITM1-R	5′-TGTCACAGAGCCGAATACCAG-3′
IFITM2-F	5′-ATCCCGGTAACCCGATCAC-3′
IFITM2-R	5′-CTTCCTGTCCCTAGACTTCAC-3′
IFITM3-F	5′-GGTCTTCGCTGGACACCAT-3′
IFITM3-R	5′-TGTCCCTAGACTTCACGGAGTA-3′
Mx1-F	5′-ACCACAGAGGCTCTCAGCAT-3′
Mx1-R	5′-CTCAGCTGGTCCTGGATCTC-3′
Mx2-F	5′-TGAACGTGCAGCGAGCTT-3′
Mx2-R	5′-GGCTTGTGGGCCTTAGACAT-3′
DDX3-F	5′-ACGAGAGAGTTGGCAGTACAG-3′
DDX3-R	5′-ATAAACCACGCAAGGACGAAC-3′
DDX5-F	5′-AGAGAGGCGATGGGCCTATTT-3′
DDX5-R	5′-CTTCAAGCGACATGCTCTACAA-3′
DDX6-F	5′-GCTGGGAAAAGCCATCTCCTA-3′
DDX6-R	5′-GGTCTAGCCGTTCAAGTAAGGG-3′
DDX21-F	5′-CTTTGCCATCCCTTTGATTGAGA-3′
DDX21-R	5′-GTAGGTGCAAGAACCAGTACC-3′
DDX50-F	5′-CTGAAAGGTCGAGGGGTAACA-3′
DDX50-R	5′-TTTGGCTACTTGGTTTGCCAG-3′
DDX60-F	5′-CAGCTCCAATGAAATGGTGCC-3′
DDX60-R	5′-CTCAGGGGTTTATGAGAATGCC-3′

### Western blotting analysis

HUVECs and A549 cells seeded in 6-well plates were mock infected or infected with HTNV at an MOI of 0.1 and then lysed at 1, 2, 3, and 4 d post-infection with 100 μl of RIPA Lysis Buffer (Beyotime, Shanghai, China) containing 50 mM Tris (pH 7.4), 150 mM NaCl, 1% NP-40, 0.5% sodium deoxycholate, 0.1% SDS and 1× protease inhibitors (Roche). The protein concentration was determined with a BCA Kit (Sigma-Aldrich). Equal amounts of protein (20 μg protein/lane) were electrophoresed in a 10% SDS polyacrylamide gel and then transferred to a PVDF membrane (Millipore, Billerica, MA, USA). After blocking with 5% bovine serum albumin (BSA, MP Biomedicals, Santa Ana, CA, USA) in Tris-buffered saline at room temperature for 1 h, the membranes were incubated with an anti-HTNV NP mouse mAb (1A8) and an anti-GAPDH mouse mAb (Protein Tech Inc.) at 4°C overnight. Anti-mouse secondary antibodies labeled with infrared dyes (LI-COR Biosciences) were used in the infrared fluorescence detection procedure. The signals on the PVDF membrane were visualized using the Odyssey Infrared Imaging System (LI-COR Biosciences).

### Flow cytometry (FCM) analysis

HUVECs infected with HTNV at an MOI of 0.1 were harvested at different time points, and FCM analysis was performed with routine protocols using a FACS Calibur flow cytometer (BD Immunocytometry Systems). FITC-conjugated 1A8 was applied for NP detection with FITC-conjugated mouse IgG1 as the isotype control. All data were analyzed using FlowJo (TreeStar).

### Immunofluorescence and confocal microscopy analysis

HUVECs cultured on glass coverslips (Millipore) until semi-confluent were incubated with HTNV at an MOI of 0.1 for 60 min. At the indicated time points post-HTNV infection, the cells were fixed with 4% PFA, incubated with 0.3% Triton X-100 (Sigma-Aldrich), blocked with 5% BSA for 1 h, and incubated with 1A8 to detect HTNV NP at 37°C for 2 h. Then, the HUVECs were washed with DPBS (HyClone) and incubated with FITC-conjugated goat anti-mouse IgG. Hoechst 33258 (100 ng/ml; Beyotime) was utilized to stain the cell nuclei, and the cells were observed using a BX60 fluorescence microscope (Olympus, Tokyo, Japan).

### Enzyme-linked immunosorbent assay (ELISA)

The HTNV titers and neutralizing antibody titers were determined by ELISA as previously described (Jiang et al., 2016). Briefly, 1A8 was coated on microplates in 0.1 M sodium carbonate bicarbonate buffer (pH 9.0) at 4°C overnight. Propagated HTNV or a sample from the neutralization test was serially diluted and incubated on the microplates at 37°C for 2 h. HRP-conjugated 1A8 was used as the detection antibody. The absorbance of the color reaction developed using TMB was measured at 450 nm. An absorbance was required and positive/negative (P/N) >2.1 was considered significant. Geometric mean titers (GMT) were utilized to compare the neutralizing activity among all groups.

### Sequencing and data processing

HUVECs were mock infected or infected with live or Co60-inactivated HTNV at an MOI of 1. RNA was extracted at 24 hpi as described above, and the quality was analyzed using the FastQC software by Beijing Genomics Institute (BGI, Shenzhen, China). Digital gene expression (DGE) tags were annotated to the human transcriptome (Ensembl Version 58) by mapping the reads to the sequences flanking the NlaIII restriction sites on both the coding and non-coding strands. Tags matching more than one gene region were discarded. The tag counts were normalized to transcripts per million (TPM) by dividing the raw tag count by the total number of tags from each library and multiplying by one million. To avoid possible signal noise from the high-throughput sequencing, genes with an average TPM of <1 in these three states were excluded.

The DGE data are available online at the Gene Expression Omnibus (GEO) under accession number GSE89950. In this study, an absolute fold change of at least 1.5 and a false discovery rate (FDR) <0.001 were used to define differentially expressed genes. Gene ontology analysis, which is organized into three independent hierarchies for cellular components, molecular functions, and biological processes, was applied to analyze the main functions of the differentially expressed genes. The significant GO terms were defined as terms with *P* < 0.05. Similarly, pathway analysis was used to identify the significant pathways of the differentially expressed genes according to KEGG, Biocarta, and Reactome (Wang et al., 2010).

### Expression of the IFITM, Mx, and DExD/H box helicase molecules

Lentiviral vectors expressing IFITM1, IFITM2, and IFITM3 were purchased from GENECHEM (Shanghai, China). The Mx1 and Mx2 expression plasmids were a kind gift from Dr. Malim MH (Goujon et al., 2013), and the DExD/H box helicase expression plasmids were kindly provided by Yasuo Ariumi (Yasuda-Inoue et al., 2013). All cDNAs were subsequently subcloned into the pLVX-IRES-ZsGreen vector (Clontech, Mountain View, CA, USA) using the Xho I and BamH I restriction sites.

To generate specific ISGs (IFN stimulated genes) or DExD/H box helicase molecule-expressing lentiviruses, pLVX-ISG was co-transfected with the lentivirus packing plasmids psPAX2 and pMD2.G (a gift from Didier Trono, Addgene plasmid #12260 and #12259) using PEI (Sigma-Aldrich) in HEK293 (293T) cells. Six hours later, the culture flask was replenished with fresh medium; the supernatants containing the lentiviruses were collected 24 and 48 h after transfection and combined. The viral titers were determined using the Lenti-X p24 Rapid Titer Kit (Clontech) according to the manufacturer's protocol. For lentiviral infection, the cells were infected with a lentivirus supplemented with 8 μg/ml of polybrene (Sigma-Aldrich) for 12 h and then incubated with fresh medium. The cells were challenged with HTNV 24 h after lentiviral infection and then fixed at 2 days post infection for ICW to detect HTNV NP expression.

### Dual-luciferase assays

The plasmids used for the luciferase assays were as follows: pGL3 basic and pRL-TK were from our lab and the reporter plasmid for the IFN-β promoter (pIFΔ (−116) lucter) was a gift from Steve Goodbourn (Childs et al., 2007). HEK293 cells were seeded into 24-well plates and transfected with the different luciferase plasmids (100 ng) and pRL-TK (10 ng). Twenty-four hours after transfection, the HEK293 cells were challenged or not challenged with HTNV at an MOI of 0.1. At 24 hpi, the cells were lysed with passive lysis buffer with gentle shaking (Promega, Madison, WI, USA). Subsequently, the Renilla and firefly luciferase activities were measured using the Dual-Luciferase Assay System (Promega). The firefly luciferase activity of the HTNV-infected samples was normalized to the Renilla activity in the same sample to control for the transfection efficiency. All experiments were performed in triplicate and repeated at least twice.

### Study participants

This study was approved by the Institutional Review Board of Tangdu Hospital, and written informed consent was obtained directly from each adult subject. Peripheral blood samples were collected from sixty-four hospitalized patients at Tangdu Hospital from October 2015 to March 2016. All patients were Han Chinese. The diagnosis of HFRS was made based on typical symptoms and signs as well as IgM and IgG antibody positivity against HTNV in the serum as assessed by ELISA by the Department of Clinical Laboratory, Tangdu Hospital. Clinical data, including the patient's age, gender, white blood cell count (WBC, × 10^9^), monocyte percentage (MONO %), heteromorphic lymphocyte rate (%), platelet count (PLT, × 10^9^), blood urea nitrogen (BUN, mmol/l), and serum creatinine (Scr, μmol/l), are shown in Table 2. The classification of HFRS severity and the exclusion criteria were previously described (Ma et al., 2012; Yi et al., 2013).

**Table 2:** Clinical and laboratory characteristics of the study population.

	**Mild/moderate patients**	**Severe/critical patients**	***P*-values**
Age (years)	42.91 ± 1.881	41.28 ± 1.549	0.5073
Gender (female/male)	10/22	8/24	0.7816
WBC (×10^9^)	14.09 ± 2.190	17.59 ± 1.418	0.1842
MONO %	10.97 ± 0.5358	9.739 ± 0.8122	0.2094
Heteromorphic Lymphocytes % lymphocytes %	9.539 ± 0.8992	10.54 ± 0.8366	0.4186
PLT (×10^9^)	106.0 ± 7.198	81.16 ± 6.535	0.0132^*^
BUN, mmol/l	10.34 ± 0.9818	22.27 ± 1.340	<0.0001^**^
Scr, μmol/l	206.6 ± 22.84	302.6 ± 26.01	0.0073^**^

*WBC, White blood cells; PLT, platelets; MONO %, monocyte percentage; BUN, blood urea nitrogen; Scr, serum creatinine. Values are presented as the mean ± SEM. P-value refers to the difference between mild and severe patients calculated using an unpaired t-test, except for the factor age (Fisher's exact test)*.

* *P < 0.05*,

** *P < 0.01 compared with the mild patients*.

Sixteen healthy individuals aged 25–35 years form the Centers for Disease Control (CDC) of Shaanxi were inoculated with inactivated bivalent vaccines for Hantaan virus (HTNV, type I) and Seoul virus (SEOV, type II) using different immune strategies (three-dose schedule at 0, 1, and 13 months). The enrolled participants were divided into two groups as follows: the vaccination without boost group (with only two primary doses of the inactivated bivalent vaccines, *n* = 8) and the boost vaccination group (three complete doses, *n* = 8). Long-term immunogenicity was evaluated by assessing the NAb titers using the ICW assay and ELISA 1 year after the final vaccination. This study was conducted in accordance with the recommendations of the biomedical research guidelines involving human participants established by the National Health and Family Planning Commission of China. The Institutional Ethics Committee of Shaanxi CDC approved this study. All subjects gave written informed consent in accordance with the Declaration of Helsinki. Prior to inclusion, all participants were informed of the study objectives and signed the consent form before medical records and blood samples were obtained.

### Statistical analysis

All data were expressed as the mean ± SD. Statistical analyses were performed using GraphPad Prism 5 (GraphPad Software, La Jolla, CA, USA). Differences among groups were determined by one-way analysis of variance (ANOVA) with repeated measures, followed by Bonferroni's *post-hoc* test. Differences between groups were analyzed with an unpaired Student's *t*-test. A nonparametric correlation (Spearman) analysis was performed to analyze associations between the ICW and ELISA-derived viral and NAb titers or between the NAb titers and other clinical parameters. *P* < 0.05 was considered significant.

## Results

### Establishment of an ICW assay to detect HTNV NP expression

**Figure 1:**
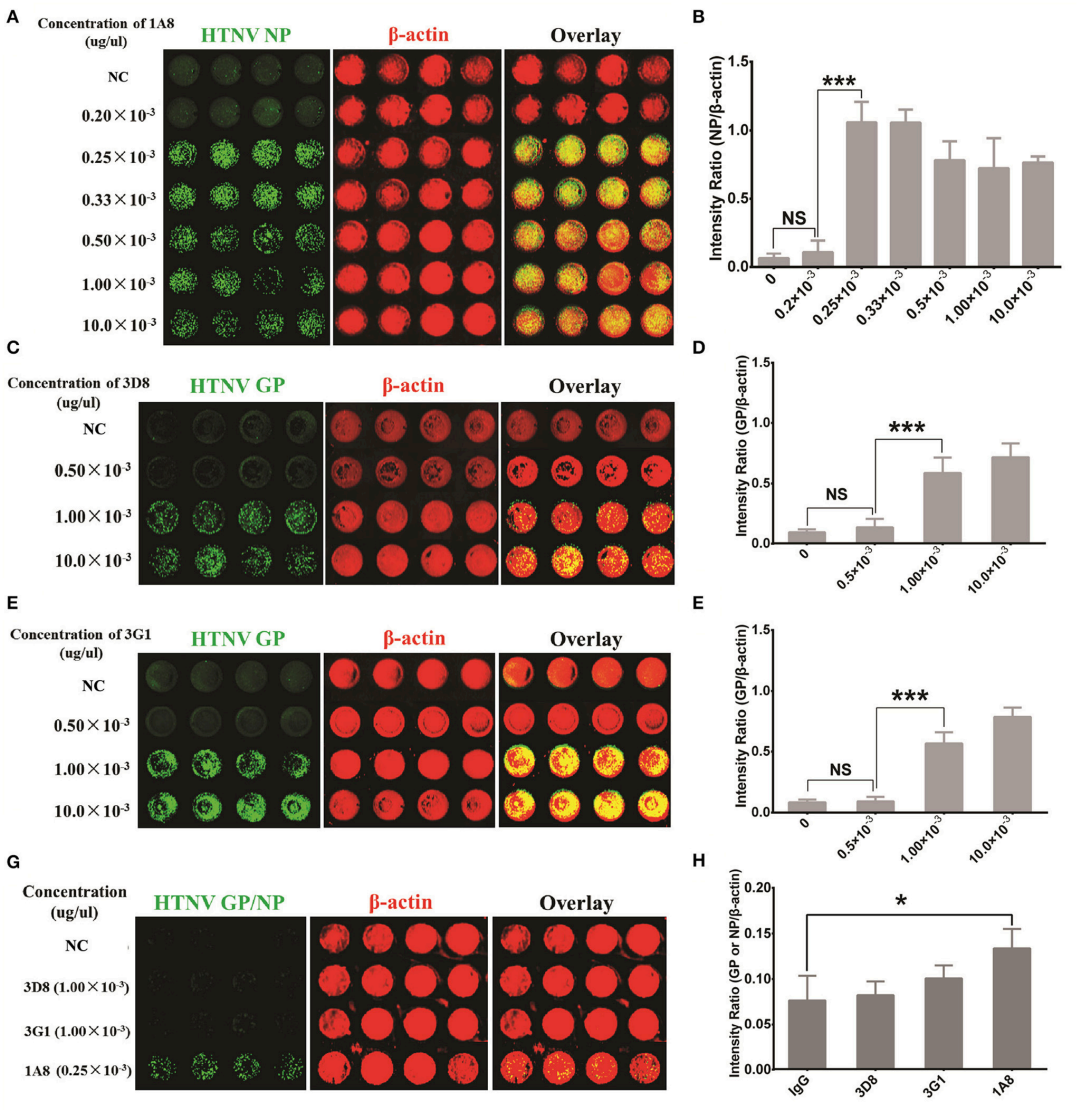
Antibody selection for HTNV NP detection with sensitivity and rapidity. HUVECs grown in 96-well microplates until they reached 60–70% confluency were infected with HTNV at an MOI of 1. The cells were fixed and evaluated with the ICW assay at 3 days post-infection **(A–F)** or 1 day post-infection **(G,H)**. **(A,B)** Mouse MAb 1A8 (1 μg/μl) were serially diluted to a final concentration of 10.0 × 10^−3^ (1:100 dilution), 1.00 × 10^−3^ (1:1,000 dilution), 0.50 × 10^−3^ (1:2,000 dilution), 0.33 × 10^−3^ (1:3,000 dilution), 0.25 × 10^−3^ (1:4,000 dilution) and 0.20 × 10^−3^ (1:5,000 dilution) (μg/μl). Sp2/0-derived mouse ascitic fluid was used as the negative control (NC). Different concentrations of 1A8 were applied in the ICW assay for HTNV NP detection. The scanned imaging results **(A)** and the intensity ratio (NP/β-actin) **(B)** are presented. **(C,D)** Serial dilutions of 3D8 targeting HTNV GP were applied in the ICW assay. The scanned imaging results **(C)** and the intensity ratio (GP/β-actin) **(D)** are presented. **(E,F)** Serial dilutions of 3G1 targeting HTNV GP were applied in the ICW assay. The scanned imaging results **(C)** and the intensity ratio (GP/β-actin) **(D)** are presented. **(G,H)** 3D8 (1.00 × 10^−3^ μg/μl), 3G1 (1.00 × 10^−3^ μg/μl) and 1A8 (0.25 × 10^−3^ μg/μl) were used in the ICW assay at 1 dpi, with Sp2/0-derived mouse ascitic fluid as the NC. The scanned imaging results **(G)** and the intensity ratio (GP or NP/β-actin) **(H)** are presented. Data are presented as the mean ± SD. ^*^P < 0.05, ^***^P < 0.001 by Student's *t*-test. The experiments were performed independently at least three times with similar results.

The first step in establishing an ICW assay to assess HTNV replication is the selection of high affinity antibodies that can sensitively detect viral protein expression. A series of mouse mAbs against HTNV proteins were prepared as previously reported (Xu et al., 2002). Of these mAbs, 1A8 had the highest affinity for the viral NP, and 3D8 and 3G1 exhibited neutralizing activity for viral Gc. These mAbs were applied in the ICW assay under identical infection conditions. As shown Figures 1A,B, HTNV NP was detected well by the ICW assay with 1A8 at concentrations higher than 0.25 × 10^−3^ μg/μl. HTNV Gc was also detected with either 3D8 (Figures 1C,D) or 3G1 (Figures 1E,F) at concentrations more than 1.00 × 10^−3^ μg/μl. Importantly, NP expression could be discerned by 1A8 at 1 day post-infection (dpi) in HUVECs, whereas Gc could not be detected by 3D8 or 3G1 at the same time point (Figures 1G,H). These data indicated that 1A8 was more sensitive and suitable for the ICW assay than 3D8 and 3G1.

**Figure 2:**
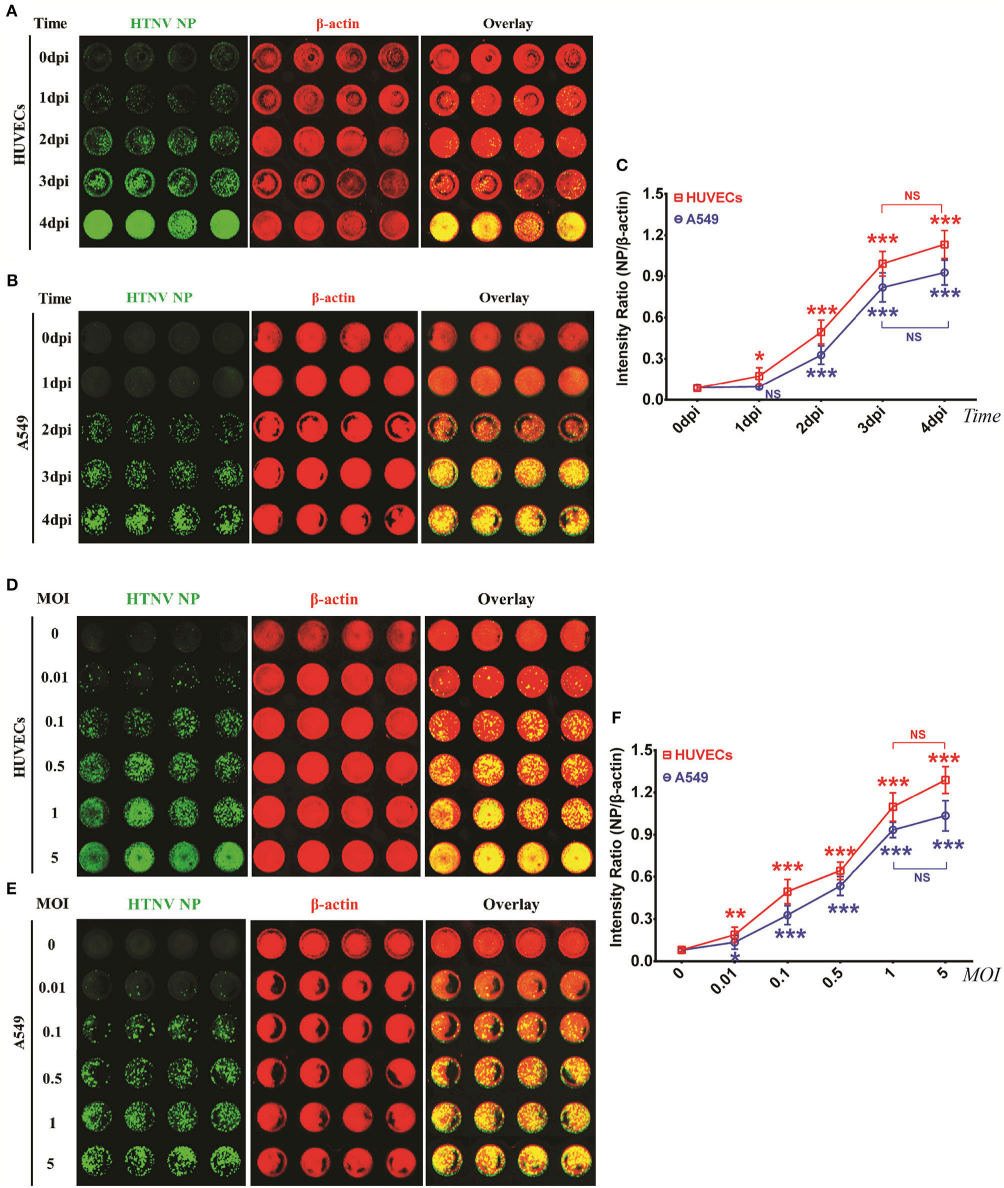
HTNV NP expression was increased in a time- and dose- depended manner in HUVECs and A549 cells within limits **(A–C)** HUVECs **(A)** and A549 cells **(B)** grown in 96-well microplates until they reached 60–70% confluence were mock infected or infected with HTNV at an MOI of 0.1 and then fixed at 0 dpi, 1 dpi, 2 dpi, 3 dpi, and 4 dpi. The ICW assay was performed with 1A8 (0.25 × 10^−3^ μg/μl) to assess HTNV NP expression. The scanned imaging results (**A** for HUVECs and **B** for A549 cells) and the intensity ratio (NP/β-actin) **(C)** are presented. All of the following experiments were performed with 1A8 (0.25 × 10^−3^ μg/μl) in the ICW assay to assess the amount of HTNV NP. **(D–F)** HUVECs **(D)** and A549 cells **(E)** grown in 96-well microplates until they reached 60–70% confluency were mock infected or infected with HTNV at incremental MOIs from 0 to 5, followed by the ICW assay performed at 2 dpi. The scanned imaging results (E for HUVECs and F for A549 cells) and the intensity ratio (NP/β-actin) **(G)** are presented. Data are presented as the mean ± SD. Differences among groups were determined by one-way analysis of variance (ANOVA) with repeated measures, followed by Bonferroni's *post-hoc* test. ^*^P < 0.05, ^**^P < 0.01, ^***^P < 0.001 vs. 0 dpi (time) or 0.01 (MOI). The experiments were performed independently at least three times with similar results.

To determine the optimal detection points, HUVECs and A549 cells were infected with HTNV and then used for the ICW assay at different time intervals. HTNV NP expression became detectable by ICW at 1 dpi in HUVECs (Figures 2A,C) or 2 dpi in A549 cells (Figures 2B,C); in both the two cell types, detection values increased from 1 dpi to 3 dpi and reached a plateau at 4 dpi (Figure 2C). These data suggested that the time point of 2 dpi was feasible for NP measurement by the ICW assay both in HUVECs and A549. To ascertain relationship between NP expression and HTNV infection doses, HUVECs and A549 cells were infected with incremental MOIs and then subjected to the ICW assay at 2 dpi. HTNV NP production was dose-dependent from an MOI of 0.01 to 5 in both the HUVECs (Figure 2D) and A549 cells (Figure 2E). Versus MOI of 0, the ICW assay could detect NP production at MOIs of 0.01, 0.1, 0.5, and 1(Figure 2F). Nevertheless, the ICW assay could not distinguish differences in NP expression between MOIs of 1 and 5 (Figure 2F). These findings indicated a clear positive relationship between NP production and the infection time or dose within limits. To efficiently and rapidly evaluate HTNV replication, the suitable infection conditions for the ICW assay are collection at 2 dpi after infection with an MOI ranging from 0.01 to 1.

### Performance comparison of the ICW assay with conventional assays

**Figure 3:**
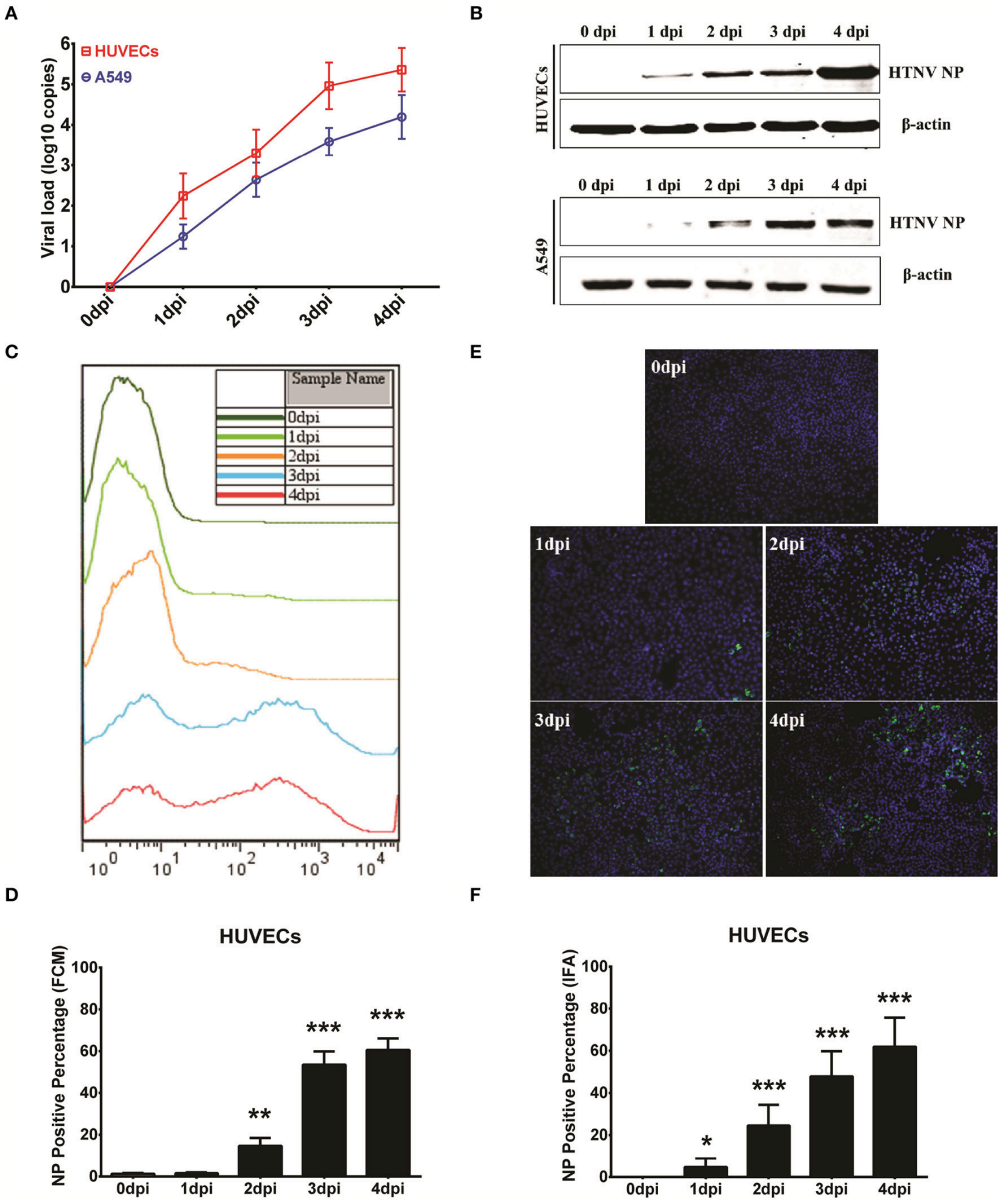
Performance comparison of the ICW assay with conventional assays. HUVECs and A549 cells grown in 6-well plate until they reached 60–70% confluence were mock infected or infected with HTNV at an MOI of 0.1 and then harvested at 0 dpi, 1 dpi, 2 dpi, 3 dpi, and 4 dpi for qRT-PCR to detect the HTNV S segment. These samples were used to calculate the viral loads **(A)** or for Western blotting to measure HTNV NP expression **(B)**. HUVECs infected at an MOI of 0.1 also underwent flow cytometry analysis **(C,D)** or an immunofluorescence assay **(E,F)** at the indicated time points post-HTNV infection. Data are presented as the mean ± SD. ^*^P < 0.05, ^**^P < 0.01, ^***^P < 0.001 vs. 0 dpi by Student's *t*-test. The experiments were performed independently at least three times with similar results.

To investigate whether detection of NP production by the ICW assay could be used to evaluate viral replication, several classical methods were performed as a reference. For this purpose, HUVECs or A549 cells were infected with HTNV at an MOI of 0.1 and then harvested at different time points post-infection. The viral loads calculated by quantification of the HTNV S segments using qRT-PCR increased from 1 dpi to 4 dpi in both the HUVECs and A549 cells (Figure 3A); this rising tendency was consistent with the detection of NP by the ICW assay (Figure 2C). The upregulated NP expression during HTNV infection was obvious by either Western blotting (Figure 3B) or the ICW assay (Figures 2A,B). These findings implied that the ICW assay was highly accurate for the evaluation of HTNV replication compared with the qRT-PCR and Western blotting assays. FCM and IFA can be used to rapidly monitor HTNV replication kinetics. Here, we found that FCM did not detect NP expression until 2 dpi in HUVECs (Figures 3C,D) and thus was less sensitive than the ICW assay (Figures 2A,C). Although, IFA could detect HTNV NP as early as 1 dpi (Figure 3E), a relatively high standard deviation was associated with this method (Figure 3F). Notably, FCM assays require an expensive apparatus, and data calculated by IFA are determined based on the selected view fields. Thus, a high-throughput screening approach is hard to establish based on FCM or IFA. These findings indicate that the ICW assay displays high accuracy and rapidity for the quantification of HTNV replication compared to the conventional methods.

### Application of the ICW assay for the detection of HTNV titers

**Figure 4:**
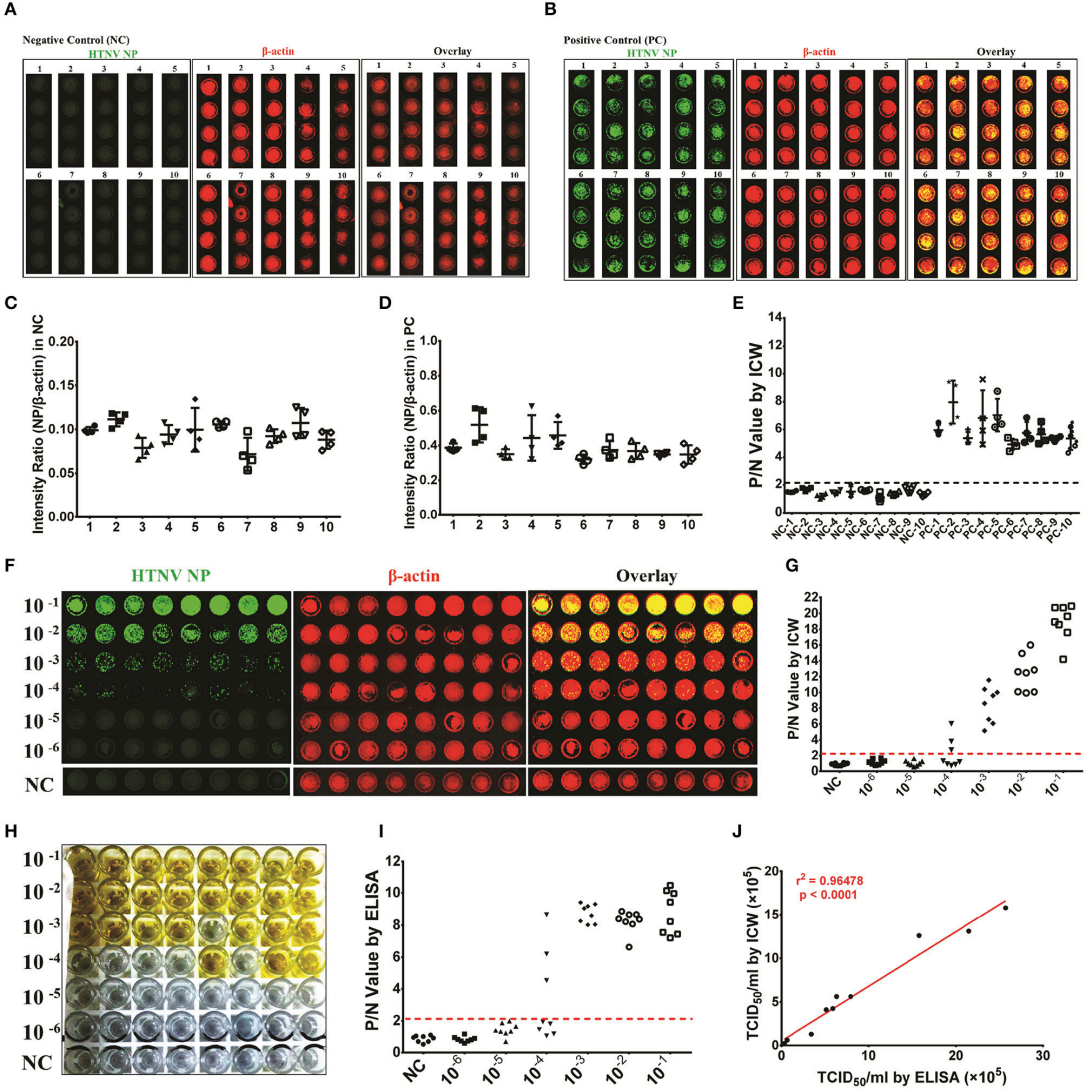
Application of the ICW assay to detect the HTNV titers HUVECs were seeded into ten 96-well microplates of different brands (Falcon™ for microplates numbered 1–5 and Nunc™ numbered 6–10) and were mock infected (negative control, NC) or infected with HTNV at an MOI of 0.1 (positive control, PC). The ICW assay was performed to observe and calculate the amount of HTNV NP production (**A** and **C** for the NC group and **B** and **D** for the PC group). The P/N value was determined by setting the intensity ratio from the number 1 microplate as the calibrator **(E)**. HTNV propagated in Vero E6 cells was serially diluted from 1:10 to 1:10^6^ and used to infect A549 cells **(F)** or E6 cells **(H)** in 96-well microplates. A549 cells were acquired for the ICW assay at 2 dpi **(F)**, and E6 cells were collected for ELISA at 10 dpi **(H)**. The P/N value was calculated by ICW **(G)** and ELISA **(I)**, and the viral titer was determined by TCID50 with the Reed and Muench formula. HTNV was propagated in mouse brains in five independent experiments and propagated in Vero E6 cells. The ten batches of HTNV were used for titer assessment by both ICW and ELISA. The relationship between the ICW-derived and ELISA-derived titers was analyzed using the rank correlation test **(J)**. Data are presented as the mean ± SD.

HTNV titers are commonly measured by TCID50 with Reed and Muench's formula through ELISA (Ye et al., 2015b). The positive or negative infection wells in the ELISA are determined by the P (positive)/N (negative) value of HTNV NP, which indicates that the negative wells have P/N values ≤ 2.1 and the positive wells have P/N values ≥ 2.1. However, the ELISA method requires at least 10 days for HTNV propagation in Vero E6 cells, which is time and labor intensive. Thus, we investigated whether the ICW assay, which only required 2 days for NP measurement, could obtain similar viral titers compared with the classical ELISA assays. A549 cells were mock infected (negative control, NC) or infected with HTNV at an MOI of 0.1 (positive control), and the ICW assay was performed at 2 dpi. To verify the repeatability of the ICW assay, both NC (Figure 4A) and PC (Figure 4B) experiments were performed ten times independently with microplates from different companies. The NP/β-actin intensity ratio in NC ranged from 0.05 to 0.15 (Figure 4C), whereas the ratio in PC fluctuated from 0.35 to 0.65 (Figure 4D). The P/N threshold value of 2.1 by ELISA was also applicable in the ICW assay (Figure 4E). Importantly, the HTNV titer calculated by the ICW assay (Figures 4F,G) was 1.26 × 10^5^ TCID50/ml, which was identical to the titer calculated using ELISA (Figures 4H,I). Next, HTNV was propagated in Vero E6 cells or mouse brains, and the titers were determined by both the ICW assay and ELISA. The results show a good linear relationship between the ICW-derived and ELISA-derived viral titers (Figure 4J), indicating that the ICW assay can be applied to assess HTNV titers.

### Application of the ICW assay to screen antiviral molecules or drugs

**Figure 5:**
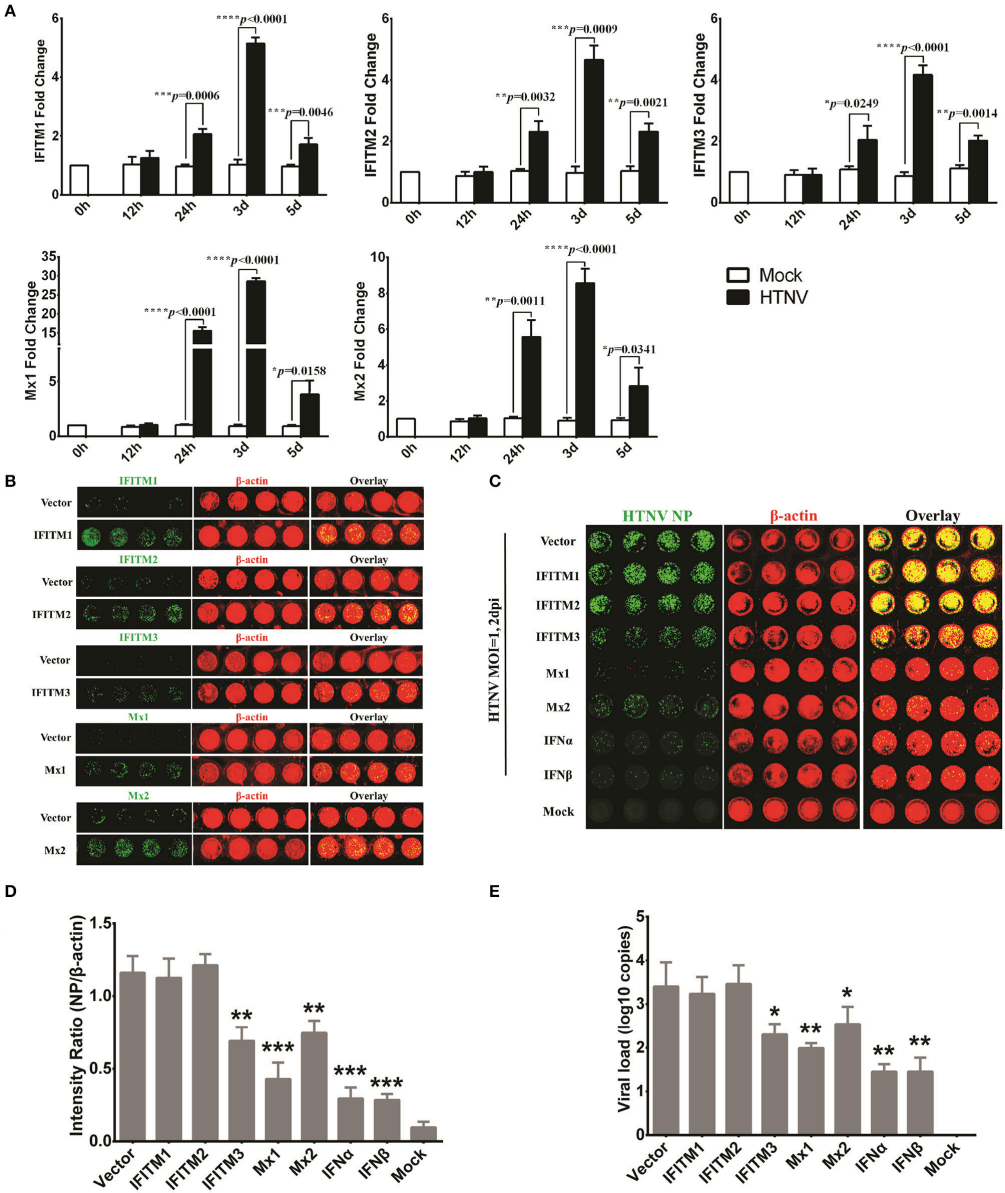
Application of the ICW assay to screen antiviral molecules or drugs. **(A)** HTNV-infected HUVECs (MOI of 1) were lysed with RNAiso at 0 h, 12 h, 24 h, 3 d, and 5 d post-infection, and the IFITM1, IFITM2, IFITM3, Mx1, and Mx2 expression levels were determined by qRT-PCR. **(B)** HUVECs transduced to express IFITM1, IFITM2, IFITM3, Mx1, and Mx2 were fixed at 3 d post-infection with the relevant lentiviruses, followed by the ICW assay to assess the overexpression of related genes. **(C–E)** HUVECs ectopically expressing IFITM1, IFITM2, IFITM3, Mx1, and Mx2 or pretreated with IFNα or IFNβ (2 ng/ml) were infected with HTNV at an MOI of 0.1. At 2 days post-infection, NP expression was measured by the ICW assay **(C,D)**, and the viral load reflected by HTNV S segment transcription was determined by qRT-PCR **(E)**. Data are presented as the mean ± SD. ^*^P < 0.05, ^**^P < 0.01, ^***^P < 0.001 vs. mock **(A)** or vector **(D,E)** by Student's *t*-test. The experiments were performed independently at least three times with similar results.

The ICW assay provides a novel approach to screen antiviral molecules or drugs and has high-throughput potential. To evaluate the utility of the ICW assay for the detection of molecules that could inhibit HTNV replication, several ISGs with definite antiviral activity but different antiviral spectra were tested in this study. We found that members of the interferon-induced transmembrane proteins (IFITMs) and dynamin-like Mx proteins were obviously upregulated at 24 h post-infection (Figure 5A) and decreased at 5 dpi. To investigate the roles of IFITM1, IFITM2, IFITM3, Mx1, and Mx2 in HTNV infection, these proteins were ectopically expressed in HUVECs, which were then infected with HTNV at an MOI of 0.1. The overexpression efficiency was confirmed by the ICW assay (Figure 5B). The amount of NP production detected by the ICW assay indicated that IFITM3 but not IFITM1 and IFITM2 significantly suppressed HTNV replication (Figures 5C,D), which was consistent as we previously reported (Xu-Yang et al., 2017). The anti-hantaviral effects of Mx1, Mx2, IFN-α, and IFN-β were also confirmed by the ICW assay (Figures 5C,D). To validate the exactitude of the ICW assay, parallel qRT-PCR experiments were performed to detect the cellular viral loads; the results were consistent with the ICW assay results (Figure 5E). These findings imply that the ICW assay can be applied to screen antiviral molecules or drugs.

### Identification of DDX21 and DDX60 as important anti-hantaviral molecules that could positively regulate IFN responses after HTNV infection

**Figure 6:**
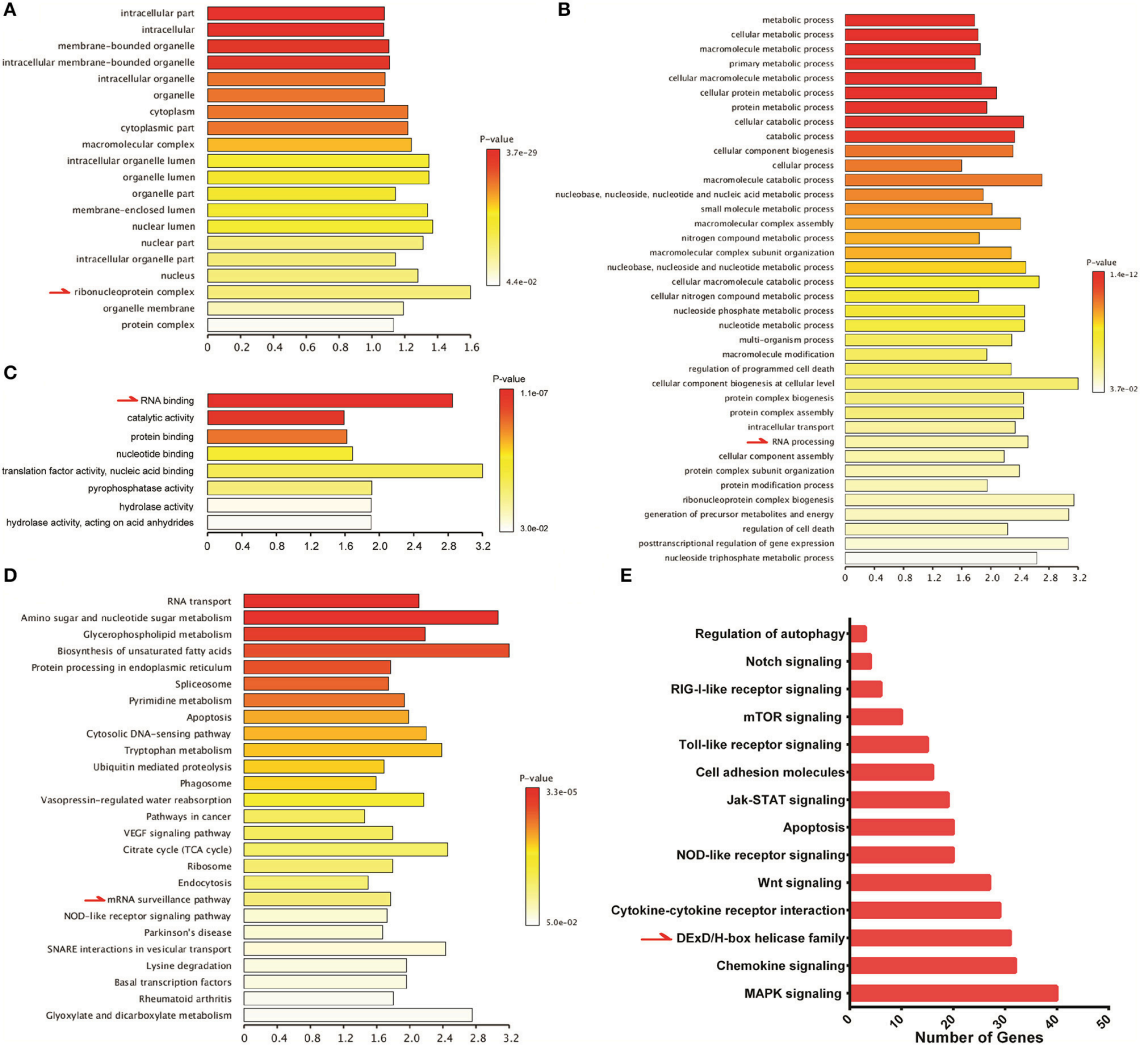
Significant gene ontology (GO) and pathway enrichment analyses of the HUVEC DGE results. Significant alterations of cellular components from the GO analysis. Significant alterations of biological processes from the GO analysis. Significant alterations of molecular functions from the GO analysis. Significant alterations of pathways based on the KEGG analysis. Enriched pathway genes from the DGE results.

**Figure 7:**
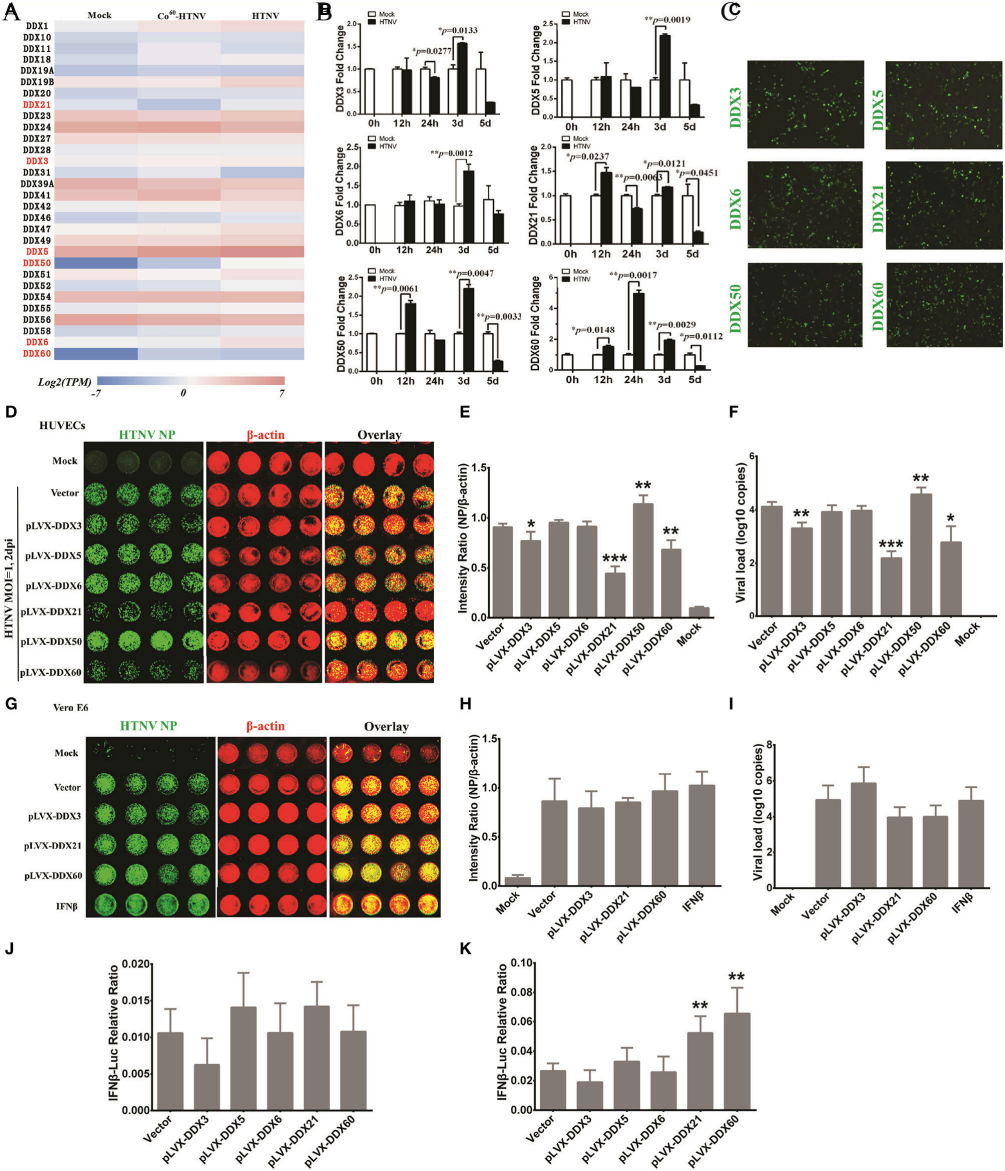
DDX21 and DDX60 were identified as important anti-hantaviral molecules that could positively regulate IFN responses after HTNV infection. **(A)** Differentially expressed DExD/H box helicases after HTNV infection shown by heat map from DGE analysis. **(B)** The altered expression levels of DDX3, DDX5, DDX6, DDX21, DDX50, and DDX60 at the different time intervals post-HTNV infection in HUVECs as determined by qRT-PCR (*n* = 3). ^*^P < 0.05, ^**^P < 0.01, ^***^P < 0.001 vs. mock by Student's *t*-test. **(C)** The overexpression efficiency of DDX3, DDX5, DDX6, DDX21, DDX50, and DDX60 in HUVECs was detected by observing the inflorescence of ZsGreen at 2 days post-infection with the related lentivirus. **(D,E)** HUVECs ectopically expressing control plasmids, DDX3, DDX5, DDX6, DDX21, DDX50, and DDX60 were mock infected or infected with HTNV at an MOI of 0.1 and then fixed at 2 dpi for the ICW assay to screen antiviral molecules. Imaging results **(D)** and the intensity ratio analyzed by software **(E)** are presented. ^*^P < 0.05, ^**^P < 0.01, ^***^P < 0.001 vs. vector by Student's *t*-test. **(F)** The viral loads of HUVECs with the identical treatment described in **(D)** were assessed by qRT-PCR (*n* = 3). ^*^P < 0.05, ^**^P < 0.01, ^***^P < 0.001 vs. vector by Student's *t*-test. **(G,H)** Vero E6 cells ectopically expressing control plasmids, DDX3, DDX21, and DDX60 or pretreated with IFN-β (2 ng/ml) were mock infected or infected with HTNV at an MOI of 0.1 and then fixed at 2 dpi for the ICW assay to screen antiviral molecules. Imaging results **(G)** and the intensity ratio analyzed by software **(H)** are presented. **(I)** Viral loads in E6 cells with the identical treatment described in **(G)** were assessed by qRT-PCR (*n* = 3). (J and K) Dual-luciferase assays of IFN-β promoter activation following HTNV infection of HUVECs. HUVECs ectopically expressing control plasmids, DDX3, DDX21, and DDX60 were transfected with 100 ng of pGL3 basic or the reporter plasmid for the IFN-β promoter (pIFΔ (−116) lucter) together with 10 ng of the pRL-TK Renilla luciferase reporter. Twenty-four hours after transfection, the HUVECs were not challenged **(J)** or were challenged **(K)** with HTNV at an MOI of 0.1 for 1 h at 37°C. Luciferase assays were performed 24 h after infection, and the results were expressed as the comparative ratio of firefly luciferase to Renilla luciferase activity compared to the untreated group. ^*^P < 0.05, ^**^P < 0.01, ^***^P < 0.001 vs. vector by Student's *t*-test. The experiments were performed independently at least three times with similar results.

To study host innate immune responses against HTNV infection, a digital gene expression (DGE) analysis was performed in HUVECs. The GO analysis suggested that increased ribonucleoprotein complex expression (Figure 6A), enhanced biological process of RNA binding (Figure 6B) and elevated molecular function RNA processing (Figure 6C) occurred after HTNV infection. The pathway involved with mRNA surveillance was remarkably activated (Figure 6D), and multiple genes belonging to the DExD/H box helicase family participated in host cellular responses against HTNV infection (Figure 6E). The DExD/H box helicases play vital roles in RNA metabolism, and some of these helicases can regulate host IFN responses and inhibit viral infection (Li et al., 2015; Diot et al., 2016). DGE analysis indicated that DDX3, DDX5, DDX6, DDX21, DDX50, and DDX60 were upregulated by HTNV (Figure 7A). Their expression levels were quantified by qRT-PCR, which showed that the enhanced expression of DDX3, DDX5 and DDX6 appeared at 3 dpi, whereas DDX21, DDX50, and DDX60 increased at 12 hpi (Figure 7B). These DExD/H box helicases were ectopically expressed in HUVECs with a lentivirus system labeled with ZsGreen (Figure 7C). Then, the cells were infected with HTNV at MOI 1. DDX3, DDX21, and DDX60 inhibited HTNV NP expression based on the ICW assay, whereas DDX50 aggravated HTNV infection (Figures 7D,E). The viral loads assessed by qRT-PCR were abated after DDX3, DDX21, or DDX60 overexpression, whereas higher HTNV loads were acquired after DDX50 overexpression (Figure 7F). Next, we investigated whether the antiviral effects of DDX3, DDX21, and DDX60 were related to IFN responses. Vero E6 cells are deficient in type I IFN production and irresponsive to type I IFN stimulation. Pretreatment with IFN-β or overexpression of DDX3, DDX21, and DDX60 did not inhibit HTNV replication in E6 cells, as shown by the ICW assay (Figures 7G,H) and qRT-PCR (Figure 7I). These findings implied that IFN responses might be necessary for the DDX3, DDX21 or DDX60-mediated antiviral influence. Indeed, overexpression of DDX3, DDX21, or DDX60 could not increase the promoter activity of IFN-β without HTNV infection (Figure 7J), whereas DDX21 and DDX60 but not DDX3 obviously promoted HTNV-induced IFN-β expression (Figure 7K). Taken together, these results indicate that DDX21 and DDX60, which were identified by the ICW assay, exert anti-hantaviral effects.

### NAb titers from patients detected by the ICW assay correlated with the HFRS disease course and severity

Micro-neutralization tests are conventionally used to assess HTNV NAb titers through ELISA, which requires at least 10 days for HTNV propagation in Vero cells.

**Figure 8:**
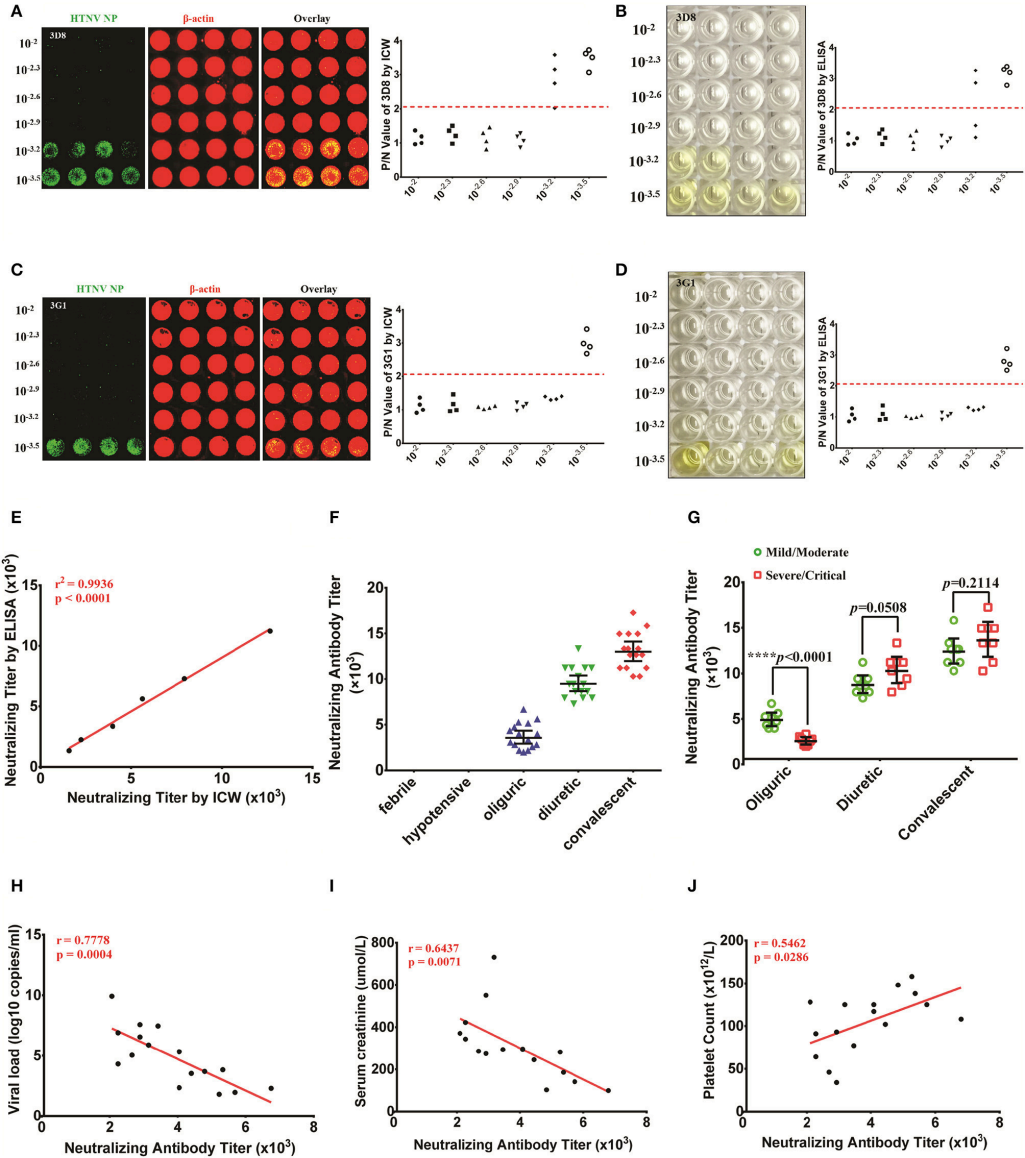
NAb titers detected by the ICW assay in patients indicated that NAbs were correlated with the HFRS disease course and severity **(A,B)** The NAb titers of 3D8 were determined in the ICW assay **(A)** and ELISA **(B)**. **(C,D)** The NAb titers of 3G1 were determined in the ICW assay **(C)** and ELISA **(D)**. **(E)** The relationship between ICW-derived and ELISA-derived NAb titers were analyzed by the rank correlation test. *P* < 0.05 were considered significant. **(F)** NAb titers of patients in different stages of HFRS were assessed using the ICW assay. Febrile (*n* = 8), hypotensive (*n* = 8), oliguric (*n* = 16), diuretic (*n* = 16), and convalescent (*n* = 16) patients were included. Data are presented as the geometric mean ± 95% CI. **(G)** The difference in NAb titers between the mild/severe and severe/critical groups at different disease stages is shown. Data are presented as the geometric mean ± 95% CI. ^*^P < 0.05, ^**^P < 0.01, ^***^P < 0.001 by Student's *t*-test. **(H,J)** Associations of the NAb titers in the oliguric stage (16 samples) with the viral load **(H)**, peak creatinine level **(I)** and peak white blood cell count **(J)** were analyzed by the rank correlation test. *P* < 0.05 were considered significant.

3D8 and 3G1 prepared by our lab had high neutralizing activity (Xu et al., 2002), with titers measured by ELISA of 1/1585 (0.0063 μg/μl) and 1/2240 (0.0045 μg/μl), respectively (Figures 8B,D). The NAb titers of 3D8 and 3G1 tested by the ICW assay at 2 dpi were 1/1334 (0.0075 μg/μl) and 1/2240 (0.0045 μg/μl), respectively (Figures 8A,C). The results indicated that NAb titers could be accurately and rapidly detected by the ICW assay. To further evaluate the ability of the ICW assay to detect NAb titers, six serum samples from HFRS patients in the oliguric or diuretic stage were collected and used to assess the NAb titers by both the ICW assay and ELISA. The results displayed a good linear relationship (Figure 8E). The findings indicate that NAb titers can be accurately and rapidly determined by the ICW assay.

Serum NAb titers from sixty-four HFRS patients were tested using the ICW assay. NAb production could not be detected in the febrile or hypotensive stage, whereas elevated NAb titers were found in the oliguric, diuretic, and convalescent stages (Figure 8F). Intriguingly, the patients in the mild/moderate group had higher NAb titers that the patients in the severe/critical group at the oliguric stage, whereas a comparison of NAb titers in the diuretic or convalescent samples from the mild/moderate group revealed no significant differences (Figure 8G). Based on these data, the relationships between the NAb titers at oliguric stage and 3 laboratory parameters that could represent the disease severity were analyzed. The data revealed a significant negative relationship between the NAb titer and the serum viral load (Figure 8H) or creatinine peak value (Figure 8I) and a significant positive relationship between the NAb titer and the lowest platelet count value (Figure 8J) in the HFRS oliguric patients.

### Efficacy evaluation of inactive HTNV vaccines by detecting NAb titers with ICW

**Figure 9:**
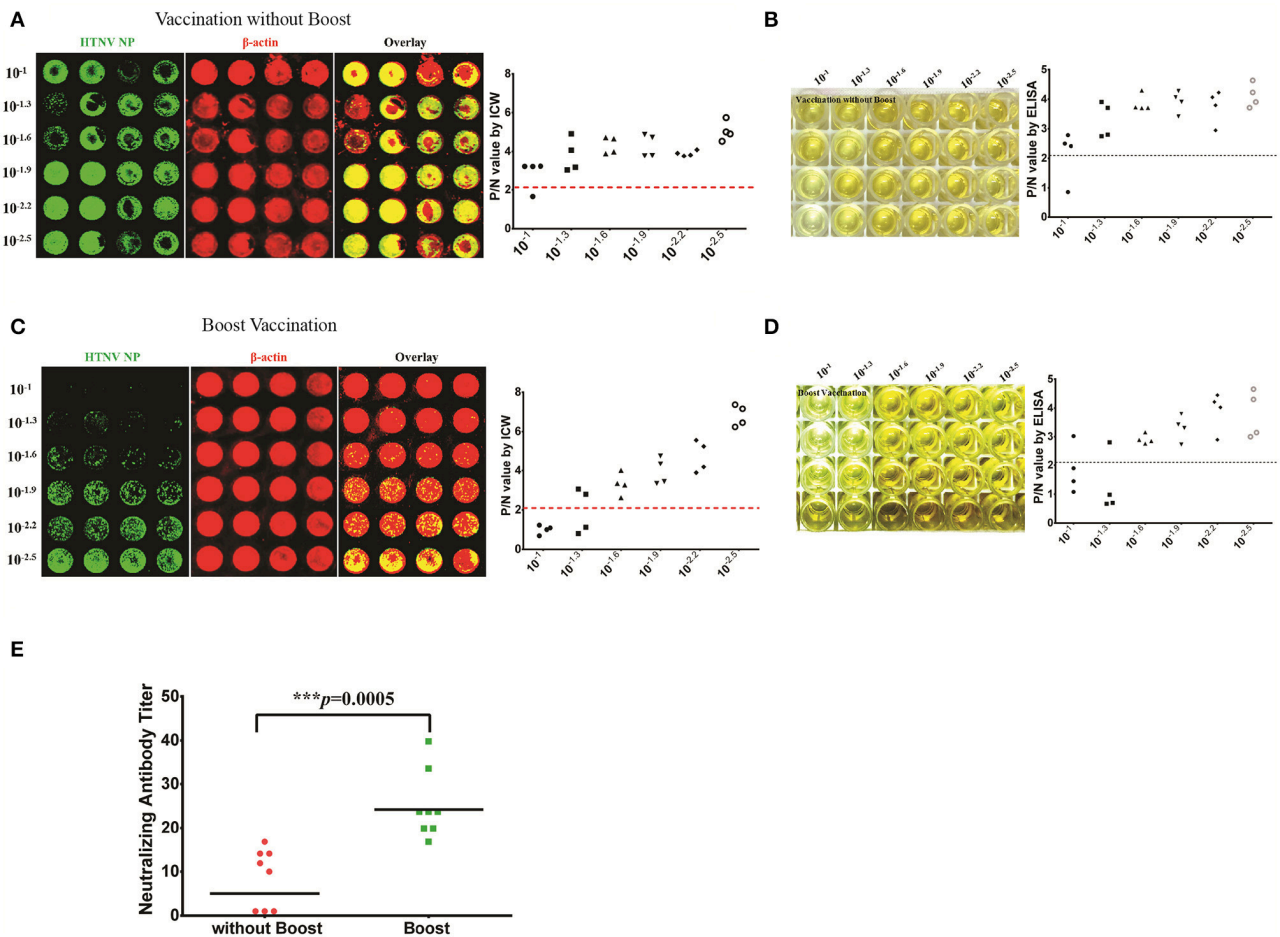
Efficacy evaluation of inactive HTNV vaccines by detecting NAb titers with ICW **(A,B)** The NAb titers of vaccinees without boost (two primary doses, *n* = 8) were determined by the ICW assay **(A)** and ELISA **(B)**. **(C,D)** The NAb titers of vaccinees in the boost group (two primary doses, *n* = 8) were determined by the ICW assay **(C)** and ELISA **(D)**. **(E)** The NAb titers of vaccinees with different vaccination strategies were assessed using the ICW assay. Notably, three individuals exhibited no production of NAbs; the NAb titers of these patients were presented as 1 to calculate the geometric mean value. Data are presented as the geometric mean ± 95% CI. ^*^P < 0.05, ^**^P < 0.01, ^***^P < 0.001 by Student's *t*-test.

NAb production reflects the host humoral immune responses against viral infection and is considered one of the most important assessment criteria for the evaluation of vaccine efficacy. The inactivated bivalent Hantaan virus (HTNV, type I) and Seoul virus (SEOV, type II) vaccines are universally applied in China to prevent HFRS. To evaluate the long-term immunogenicity of the bivalent inactivated hantavirus vaccine, sixteen healthy vaccinees aged from 18 to 50 years were enrolled and divided into the vaccination without boost group (two primary doses, *n* = 8) and the boost vaccination group (booster administration, *n* = 8). The serum NAb titer of one individual from the vaccination without boost group was 1/8 as detected by the ICW assay (Figure 9A) and ELISA (Figure 9B). The serum NAb titer of one individual from the boost vaccination group was 1/20 as detected by the ICW assay (Figure 9C) and ELISA (Figure 9D). Although boost vaccination enhanced NAb production (Figure 9E), the NAb titers of the vaccinees were significantly lower than the titers of individuals recovering from HFRS (Figures 9E, 8F), indicating that the current vaccine still required further improvement to efficiently prime host humoral immunity.

## Discussion

Hantaan virus (HTNV) is the major pathogenic agent of HFRS in China and other Asian countries. As a potential weapon for bioterrorism, HTNV represents an immense threat for global public health and safety. The current ineffective therapeutic and prophylactic strategies against HTNV infection require studies investigating the development of valid anti-hantaviral drugs and vaccines, while the latter calls for rapid detection of viral titers. Nevertheless, the non-lytic characteristic of HTNV frustrates the classical plaque assays used to assess the viral titers. Here, to tackle this problem, we applied an ICW assay to establish a novel approach to evaluate HTNV replication and more precisely calculate the viral titers. Furthermore, a series of antiviral molecules from the DGE analysis were screened, and NAb titers were measured in HFRS patients and vaccine recipients.

The NP production of hantaviruses is used to quantify the amount of infectious particles and reflect the viral replication level (Koma et al., 2012; Barriga et al., 2013; Guo et al., 2015; Cheng et al., 2016; Ma et al., 2016). Compared with conventional methods, such as ELISA, the ICW assay promptly detected HTNV NP expression, and the results were characterized by high accuracy and quality. Notably, the NP spotted and exhibited in the ICW results forms obvious stains that mimic plaque-forming units. However, the specific values scanned and analyzed by the ICW assay only reflect the fluorescence intensity of the integral well instead of the number of spots. As a consequence, the intensity represented the quantity of NP production but could not directly indicate the virulence, which was better shown by plaque-forming assays. For viral titer assessment, the TCID50 detected by the ICW assay was more convenient and rapid than the ELISA-based TCID50 assay. The ICW-based viral titer detection method can also be applied in other non-CPE hantaviruses, such as Puumala virus and Dobrava-Belgrade virus. Additionally, the titer of other kinds of non-CPE or poorly-lytic viruses, such as HCV, can also be measured by detecing core protein or other viral proteins with ICW. To screen antiviral molecules, the ICW results could be repeated using other methods. Some IFN stimulated genes (ISG), such as IFITM3, Mx1, Mx2, and the type I IFNs, were confirmed to exert anti-hantaviral effects by the ICW assay. Thus, the well-established ICW assay offers a novel platform for screening antiviral molecules and developing therapeutic drugs.

Using DGE and GO analysis, we found that DExD/H box helicase family members most likely regulated host immune responses against HTNV infection. DExD/H box helicases are highly conserved and expressed in cells to regulate RNA metabolism by unwinding RNA structures or dissociating RNA-protein complexes (Fullam and Schroder, 2013). DExD/H-box helicases have been reported to play dual roles during viral infection. Some members belonging to the DExD/H-box helicase family can act as either the sensor to detect viral nucleic acids and enhance host innate immune responses or the effector to directly clear the viruses (Ahmad and Hur, 2015). Additionally, several DExD/H-box helicases facilitate viral replication and are considered valuable targets for the development of a broad spectrum of antiviral agents (Brai et al., 2016). The RIG-like helicases were confirmed as vital cytosolic pattern recognition receptors for viral RNA because their activation led to the induction of type I interferons and pro-inflammatory cytokines (Oshiumi et al., 2016). DDX3 promoted stress granule formation and inhibited influenza virus infection (Thulasi Raman et al., 2016). Nevertheless, DDX3 was considered a host factor that was hijacked by viruses and favored the replication of flaviviruses (e.g., HCV, dengue virus and West Nile virus) and retroviruses (e.g., HIV-1; Brai et al., 2016). Here, we found that DDX3 was upregulated by HTNV and that DDX3 overexpression suppressed HTNV replication in an IFN-independent manner. DDX5 combined with the 3′ untranslated region of Japanese encephalitis virus (JEV) increased JEV replication (Li et al., 2013). DDX6 constitutes the P-body in host cells, which is exploited by flaviviruses (e.g., HCV, dengue virus and West Nile virus) and retroviruses (e.g., HIV-1) for their replication (Jangra et al., 2010; Ward et al., 2011; Reed et al., 2012; Chahar et al., 2013). Although, HTNV infection promoted DDX5 and DDX6 expression, neither of these helicases had a significant influence on HTNV infection in our study. DDX21 and DDX60 interact with TRIF and RIG-I, respectively, to assist with dsRNA recognition and boost IFN production (Miyashita et al., 2011; Zhang et al., 2011; Tsai et al., 2014; Grunvogel et al., 2015; Oshiumi et al., 2015). However, the antiviral spectra of DDX21 and DDX60 are obscure. Overexpression of DDX21 and DDX60 exerted anti-hantaviral effects in an IFN-dependent manner, as indicated by the ICW approach. DDX50 contains genomic structures similar to DDX21 (Ohnishi et al., 2009; Siatelis et al., 2011), but the function of DDX50 remains a mystery. We found that DDX50 might be as a positive regulator of HTNV replication, indicating that DDX50 harbored opposite effects against DDX21 upon viral infection.

Different DExD/H helicases induced by HTNV infection exhibited unequal alterations. The levels of DDX3, DDX5, DDX6, DDX21, and DDX50 were elevated by <2.5-fold, whereas the DDX60 level was elevated by ~6-fold; these results implied that the overexpression of these helicases was more meaningful than silencing them. We used Vero E6 cells, which are deficient in IFN responses, to investigate whether the antiviral activity of DDX3, DDX21, and DDX60 depended on IFN production. Although these DExD/H helicases have high conservation between humans and monkeys, ectopically expressed human-derived DDX3, DDX21, or DDX60 may not function in monkey Vero E6 cells.

Neutralizing antibodies (NAbs) efficiently block viral infection. NAb production influences the outcome of infectious diseases and is an important criterion for vaccine efficacy. In our study, the NAb titers of HFRS patients and vaccinated individuals were tested using the ICW assay. High NAb levels in the patients were detected in the oliguric, diuretic and convalescent stages of HFRS. During the oliguric stage of HFRS, patients in the mild/moderate group showed higher NAb production than patients in the severe/critical group. The NAb titers were correlated with the serum viral load, the peak creatinine concentration and the lowest platelet count. These findings indicated that patients with prompt NAb production tended to have a favorable prognosis. Additionally, NAb titers against HTNV in the vaccine recipients, even those boosted with three doses, were extremely low compared to the convalescent patients, the results of which were consistent with the recent analysis of the long-term immunogenicity of the inactivated Hantaan virus vaccine in healthy adults (Song et al., 2016). To acquire effective protection, the NAb titers should be about 1/100 as we reported in animal experiments (Ying et al., 2016). Obviously, NAb titers against HTNV in the vaccine recipients were much lower than 1/100, indicating that those recipients still have high risk for HTNV infection. The data indicated that the current inactivated bivalent vaccines in China still needed further improvements to efficiently activate host humoral immunity.

In summary, our study applied the ICW assay to detect viral protein expression, thereby establishing a novel approach to evaluate viral replication and detect viral titers, especially targeted at non-lytic viruses such as HTNV. Using the ICW assay, we demonstrated for the first time that DDX21 and DDX60 exerted anti-hantaviral effects in an IFN-dependent manner, whereas DDX50 promoted HTNV replication. Importantly, we discovered that HFRS patients produced high NAb levels since the oliguric stage and that rapid NAb production negatively correlated with the HTNV load and disease severity. We also confirmed the poor effects of the current vaccine on NAb stimulation. These results provide new insights into the application of the ICW assay for the assessment of viral titers and NAb titers. Thus, this method can be used to identify new targets for the development of novel agents against HTNV infection and evaluate vaccine efficacies.

## Author contributions

HM, WY, and HC are responsible for the contents of the main experiments and the writing the manuscript. TN and LC are responsible for the flow cytometry and partial molecular cloning experiments. LZ and PH are responsible for the clinical sample and data collection. XW and ZX are responsible for experimental instruction. YL and FZ are responsible for the experimental design. All authors read and approved the final manuscript.

### Conflict of interest statement

The authors declare that the research was conducted in the absence of any commercial or financial relationships that could be construed as a potential conflict of interest.
